# Metabolic risk stratification and psychotic symptoms in first-episode drug-naïve major depressive disorder: a principal component-based cross-sectional study

**DOI:** 10.3389/fpsyt.2026.1916151

**Published:** 2026-08-25

**Authors:** Miao Chen, Xiaofang Shang, Xiao-E Lang, Junjun Liu, Xiangyang Zhang

**Affiliations:** 1 Department of Psychiatry, The Affiliated Brain Hospital of Nanjing Medical University, Nanjing, China; 2 Department of Psychiatry, First Hospital of Shanxi Medical University, Taiyuan, China; 3 Department of Psychiatry, Nanjing Meishan Hospital, Nanjing, China; 4 Hefei Fourth People’s Hospital, Anhui Mental Health Center, Affiliated Psychological Hospital of Anhui Medical University, Hefei, China

**Keywords:** chain mediation analysis, glucose-lipid metabolism, major depressive disorder, metabolic risk stratification, psychotic symptoms

## Abstract

**Objective:**

To examine the association between glucose-lipid metabolism and psychotic symptoms in first-episode drug-naïve (FEDN) major depressive disorder (MDD) patients.

**Methods:**

A single-center cross-sectional study enrolled 1718 FEDN MDD patients (171 with, 1547 without psychotic symptoms). Fasting blood glucose, lipid profiles, body mass index, and HAMD, HAMA, PANSS scores were collected. Principal component analysis (PCA) reduced collinearity, serial association analysis explored association patterns, K-means clustering stratified metabolic risk, and multivariate logistic regression confirmed independent associations.

**Results:**

Two metabolic components were extracted by PCA. The cholesterol-glucose-related first component (PC1) was most closely linked to psychotic symptoms. Cross-sectional path analysis showed that the statistical covariance between PC1 and psychotic symptoms was predominantly accounted for by the severity gradients of depressive and anxiety symptoms, with total indirect association 0.0440 (95% CI: 0.0361 - 0.0533) and no significant direct association. Participants were classified into three metabolic risk subgroups by PC1 scores. Prevalence of psychotic symptoms increased progressively across the low, medium, and high risk groups (0.61%, 3.44%, and 32.23%, respectively). Multivariate logistic regression validated metabolic risk as an independent predictor of psychotic symptoms, with adjusted ORs 0.076 (medium-risk) and 0.013 (low-risk) (all P < 0.001). Receiver operating characteristic analysis also showed its discriminative ability.

**Conclusion:**

Cholesterol-glucose metabolic alterations are associated with psychotic symptoms via sequential depressive and anxiety symptoms in FEDN MDD patients.

## 1 Introduction

Major depressive disorder (MDD) constitutes a major global public health challenge. It affects around 229 million people across different geographic regions (1). It is a leading cause of years lived with disability, ranking second among all major disabling conditions and contributing significantly to the global disease burden (1). Severe MDD greatly increases the consumption of outpatient medical resources and leads to substantial work absence (2). Psychotic depression (PD) is a particularly severe subtype of MDD characterized by the presence of delusions or hallucinations during depressive episodes. Patients with this subtype experience worse long-term outcomes, including higher rates of recurrence, increased hospitalization risk, and greater challenges in clinical management compared to those with non-psychotic depression (3). It differs distinctly from non psychotic depression in treatment strategies and prognostic outcomes (4). Regrettably, psychotic symptoms in MDD patients are often overlooked in clinical practice, which easily results in missed diagnosis and inadequate treatment, and ultimately brings adverse clinical consequences (3).

The prevalence of PD varies markedly across different populations and studies due to inconsistent diagnostic criteria and assessment approaches (5, 6). Relevant epidemiological data show that its prevalence is about 0.4% in the general population of Europe, while the proportion of psychotic features ranges widely among community residents, outpatient visitors, and inpatients with MDD (5, 6). In clinical practice, there is no unified consensus on the diagnostic positioning of PD, which further causes divergent prevalence estimates in published literature (6, 7). At present, the specific pathological mechanisms underlying PD remain poorly understood, and reliable objective biomarkers for early identification are still lacking. Although several studies have found potential correlations between brain functional changes, neurotransmitter disturbance, and PD onset, the complete action mechanisms have not been fully elaborated, and mature biomarker systems are yet to be established (8, 9).

Accumulating genetic and clinical evidence has confirmed the close link between depressive disorders and metabolic dysfunctions (10, 11). Genetic analyses reveal that depression is closely related to elevated metabolic syndrome risk and abnormal levels of multiple metabolic indicators (11). Clinical observations in drug-naïve patients with first episode MDD further prove that PD patients are more vulnerable to early metabolic disorders (10). They have a much higher incidence of metabolic syndrome and present obvious disturbances in blood glucose and blood lipid profiles when compared with patients without psychotic symptoms (10). Nevertheless, traditional relevant studies have notable methodological flaws. These studies fail to address collinearity among metabolic indicators, cannot distinguish between causal relationships and simple correlations, and are unable to identify heterogeneous patient subgroups, making it difficult to translate their findings into individualized clinical intervention strategies.

To address these research gaps, we developed a comprehensive three-level statistical analysis system and employed three complementary analytical methods in the present study. We selected five core metabolic indicators, including fasting glucose and multiple lipid indices, as research variables, and also incorporated body mass index (BMI), which is easily obtained and can well reflect overall metabolic status, for principal component analysis (PCA) (12, 13). This dimensionality reduction method effectively eliminates collinearity interference, helping to identify core metabolic features related to psychotic symptoms. We further applied serial association analysis to clarify the internal causal pathways between metabolic abnormalities and psychotic symptoms, and adopted cluster analysis to complete patient risk stratification. Such a research design can effectively translate population-based research findings into practical tools for individualized clinical evaluation.

On the basis of the above methodological framework, we formally propose three testable research hypotheses. Given the cross-sectional design of this study, we focus on examining the patterns of association between variables. First, FEDN MDD patients with psychotic symptoms exhibit distinct glucose and lipid metabolic profiles compared to those without psychotic symptoms. Second, the association between metabolic dysregulation and psychotic symptoms is primarily explained by the sequential co-occurrence of depressive and anxiety symptoms, rather than a direct link between metabolic factors and psychotic manifestations. Third, risk stratification based on core metabolic features can effectively identify subgroups of FEDN MDD patients with different prevalence rates of psychotic symptoms, and the prevalence of psychotic symptoms increases with higher levels of metabolic abnormality.

## 2 Methods

### 2.1 Study procedure and participants

We conducted this single-center cross-sectional study between 2015 and 2017 at the Department of Psychiatry, First Clinical Medical College of Shanxi Medical University, Taiyuan, China. We consecutively recruited outpatients with first-episode drug-naïve major depressive disorder (FEDN MDD). We enrolled Han Chinese participants aged 16 to 60 years who met the following criteria. Two board-certified psychiatrists independently confirmed a primary diagnosis of MDD using the Chinese version of the Structured Clinical Interview for Diagnostic and Statistical Manual of Mental Disorders Fourth Edition (DSM-IV) Axis I Disorders (14). All participants presented with an acute depressive episode and had a 17-item Hamilton Rating Scale for Depression (HAMD-17) score of at least 24 at baseline (15). None had received antidepressants, antipsychotics or any other psychotropic medications prior to enrollment. We limited illness duration to 2 years or less to minimize the impact of chronic disease progression on metabolic profiles. Exclusion criteria included other DSM-IV Axis I disorders, clinician-confirmed severe physical illness, substance abuse/dependence (except tobacco smoking), pregnancy/lactation, and inability or refusal to provide written informed consent.

The study protocol was approved by the Institutional Review Board (IRB) of the First Clinical Medical College of Shanxi Medical University (Approval No. Y27). All potential participants received a full explanation of the study before any study-related procedures, and all included subjects voluntarily provided written informed consent.

### 2.2 Measures

#### 2.2.1 Basic information

We used a self-designed questionnaire during clinical interviews to collect demographic and clinical data from all participants. Demographic data included age, gender (male/female), marital status (unmarried/married), education level (middle school or below, high school, college, or postgraduate), height, and weight. All data were cross-checked against medical records to ensure accuracy.

#### 2.2.2 Clinical symptoms measures

We assessed clinical symptoms of all enrolled participants using three well-validated psychiatric rating scales, all of which have been verified for reliable application in Chinese populations. We assessed depressive symptom severity using the validated Chinese version of the 17-item HAMD. Eight items were scored from 0 to 4 based on symptom intensity, while the remaining nine items used a 0 to 2 rating scale (15). Anxiety symptoms were evaluated with the 14-item Hamilton Anxiety Rating Scale (HAMA), which employs a uniform 0 to 4 scoring system for all items (16). We used the positive symptom subscale of the Positive and Negative Syndrome Scale (PANSS) to quantify psychotic manifestations (17). We adopted this 7-item subscale which uses a standardized 7-point Likert scale. A score of 1 corresponds to absent symptoms, while 7 indicates the most severe presentation. The PANSS positive subscale ranges from 7 to 49, with a cutoff of ≥15 applied to define psychotic symptoms, consistent with previous studies (17–19).

All assessments were completed independently by two trained psychiatrists who received standardized pre-study training on scale administration and scoring. The inter-rater intraclass correlation coefficient (ICC) for all three scale total scores remained above 0.8 throughout the study, and raters were blinded to participants’ clinical data and study grouping to minimize measurement bias.

#### 2.2.3 Biochemical measurements

We collected fasting venous blood samples from all enrolled participants between 6:00 and 8:00 a.m. after an overnight fast of at least 8 hours. All samples were immediately sent to the hospital’s central clinical laboratory, with all biochemical tests completed before 11:00 a.m. on the sampling day. Measurements were performed under standardized laboratory protocols with strict internal quality control, and the tested parameters included fasting blood glucose (FBG), total cholesterol (TC), triglycerides (TG), low-density lipoprotein cholesterol (LDLC), and high-density lipoprotein cholesterol (HDLC).

### 2.3 Statistical analysis

We performed all statistical analyses using R software version 2022.07.0. A two sided P value less than 0.05 was set as the threshold for statistical significance. Continuous variables were expressed as mean ± standard deviation. Categorical variables were reported as counts and corresponding percentages. Between group comparisons were conducted with independent samples t tests for continuous data and chi square tests for categorical data. We implemented a three tiered analytical strategy to address limitations of prior studies. First, we conducted PCA on six metabolic parameters. These parameters included BMI calculated as weight in kilograms divided by height in meters squared, FBG, TC, TG, LDLC and HDLC. This dimensionality reduction approach aimed to extract core metabolic features and mitigate multicollinearity among correlated indicators. All variables were standardized to z scores prior to analysis. We retained components based on the Kaiser Guttman rule and parallel analysis. Factor loadings of 0.3 or higher were considered statistically meaningful. We used Pearson correlation analysis to evaluate associations between the extracted principal components and clinical symptom scores. Only the component with the largest variance contribution was selected for subsequent analyses. This approach helped control for multiple comparison bias. We included all six metabolic indicators in the principal component analysis. No individual metabolic parameter was adjusted separately to avoid introducing multicollinearity into subsequent models.

We next performed serial association analysis using the lavaan package in R to delineate the sequential covariance structure linking metabolic traits to psychotic symptoms (20). The principal component score derived from the above analysis was designated as the independent variable. Depressive and anxiety symptoms served as sequential intermediate factors. Psychotic symptoms were treated as the binary outcome. Participants with a PANSS positive subscale score of 15 or higher were classified as having psychotic symptoms and coded as 1. Those with lower scores were coded as 0. We conducted significance testing using bias corrected bootstrapping with 5000 resamples. All models were adjusted for predefined confounding variables. Third, we applied K-means clustering to explore the stratification of participants into distinct metabolic risk subgroups based on the preselected principal component. The optimal number of clusters was determined by a combination of the elbow method, silhouette coefficient, and clinical interpretability, with the three-cluster solution selected as the most clinically meaningful and parsimonious grouping. We then used multivariate logistic regression to examine the independent association between metabolic risk stratification and the presence of psychotic symptoms. Multicollinearity among covariates was assessed using variance inflation factors (VIF), with a threshold of 5 indicating substantial collinearity. Covariates in the regression model included age, gender, education level and marital status. These variables were selected based on univariate comparison results and established clinical relevance. Individual metabolic parameters were not included as separate covariates to avoid multicollinearity with the composite metabolic dimension derived from PCA, as these indicators may be highly correlated with the extracted principal components by design. No individual metabolic parameters were included in the regression model. This was consistent with our pre-specified statistical analysis plan. To explore potential effect modification by sex, we included an interaction term between sex and the extracted principal component score in an additional logistic regression model, with stratified analyses performed for males and females separately. No individual metabolic parameters were included in the regression model. This was consistent with our pre specified statistical analysis plan. To evaluate the discriminant validity of the PANSS positive subscale and to quantify its overlap with affective symptom severity, we performed Pearson correlation analyses among the total scores of the PANSS positive subscale, HAMD, and HAMA.

To evaluate the discriminative ability of the extracted metabolic principal components for psychotic symptoms, we performed receiver operating characteristic (ROC) curve analysis. The area under the curve (AUC) was calculated for the extracted principal components and each individual metabolic parameter. An AUC greater than 0.5 indicated discriminative ability beyond chance. The ROC analysis was conducted using the pROC package in R.

## 3 Results

### 3.1 Demographic and clinical characteristics

Our study included 1718 FEDN MDD patients, divided into the psychotic symptoms group (n = 171) and the non-psychotic symptoms group (n = 1547). No significant differences were found in age (P = 0.0535), sex distribution (P = 0.0611) and marital status (P = 0.9342) between the two groups. Patients with psychotic symptoms had significantly higher BMI (24.67 ± 1.90 vs 24.33 ± 1.92 kg/m², t = 2.22, P = 0.0275) (**Table 1**).

**Table 1:** Demographic and clinical characteristics of major depressive disorder patients grouped by psychotic symptoms.

Variables	PD (n = 171)	NPD (n = 1547)	t/χ²	P	Significance
Age (years, M ± SD)	36.7661 ± 13.5878	34.6587 ± 12.2816	1.9423	0.0535	NS
BMI (kg/m2, M ± SD)	24.6739 ± 1.904	24.3331 ± 1.9246	2.2191	0.0275	*
FBG (mmol/L, M ± SD)	5.6754 ± 0.7359	5.3665 ± 0.6285	5.281	0	***
TC (mmol/L, M ± SD)	5.7513 ± 1.1587	5.1939 ± 1.0854	6.007	0	***
TG (mmol/L, M ± SD)	2.4526 ± 0.9659	2.1358 ± 0.9839	4.063	0.0001	***
LDLC (mmol/L, M ± SD)	3.2181 ± 0.8509	2.9581 ± 0.8568	3.7894	0.0002	***
HDLC (mmol/L, M ± SD)	1.1508 ± 0.2822	1.2275 ± 0.2879	-3.3655	0.0009	***
HAMD score	33.8655 ± 2.63	29.9024 ± 2.7	18.6485	0	***
HAMA score	26.3743 ± 3.0137	20.181 ± 2.9288	25.5712	0	***
PANSS positive subscale score	21.3743 ± 2.6502	7.4667 ± 1.191	67.8685	0	***
Sex, n (%)			3.5073	0.0611	NS
Male	47 (27.5%)	541 (35%)			
Female	124 (72.5%)	1006 (65%)			
Marital status, n (%)			0.0068	0.9342	NS
Unmarried	49 (28.7%)	453 (29.3%)			
Married	122 (71.3%)	1094 (70.7%)			
Education level, n (%)			11.9118	0.0077	**
Middle school or below	59 (34.5%)	354 (22.9%)			
High school	69 (40.4%)	691 (44.7%)			
College	35 (20.5%)	414 (26.8%)			
Postgraduate	8 (4.7%)	88 (5.7%)			

BMI, body mass index; FBG, fasting blood glucose; HAMA, Hamilton anxiety rating scale; HAMD, Hamilton depression rating scale; HDL-C, high-density lipoprotein cholesterol; LDL-C, low-density lipoprotein cholesterol; M, mean; n, number; NPD, non-psychotic depression (PANSS positive subscale score ≤ 14); NS, not significant; PANSS, positive and negative syndrome scale; PD, psychotic depression (PANSS positive subscale score ≥ 15); SD, standard deviation; TC, total cholesterol; TG, triglycerides; *, P < 0.05; **, P < 0.01; ***, P < 0.001.

All measured fasting glucose and lipid parameters showed significant between-group differences (all P < 0.001) (**Table 1**). Patients in the psychotic symptoms group had higher levels of fasting blood glucose (5.68 ± 0.74 vs 5.37 ± 0.63 mmol/L, t = 5.28), total cholesterol (5.75 ± 1.16 vs 5.19 ± 1.09 mmol/L, t = 6.01), triglycerides (2.45 ± 0.97 vs 2.14 ± 0.98 mmol/L, t = 4.06) and low-density lipoprotein cholesterol (3.22 ± 0.85 vs 2.96 ± 0.86 mmol/L, t = 3.79), and lower levels of high-density lipoprotein cholesterol (1.15 ± 0.28 vs 1.23 ± 0.29 mmol/L, t = -3.37).

Patients with psychotic symptoms also had significantly higher scores on all clinical symptom scales (all P < 0.001) (**Table 1**). Their HAMD score (33.87 ± 2.63 vs 29.90 ± 2.70, t = 18.65), HAMA score (26.37 ± 3.01 vs 20.18 ± 2.93, t = 25.57) and PANSS positive subscale score (21.37 ± 2.65 vs 7.47 ± 1.19, t = 67.87) were all significantly elevated compared with patients without psychotic symptoms.

### 3.2 Principal component analysis

**Figure 1:**
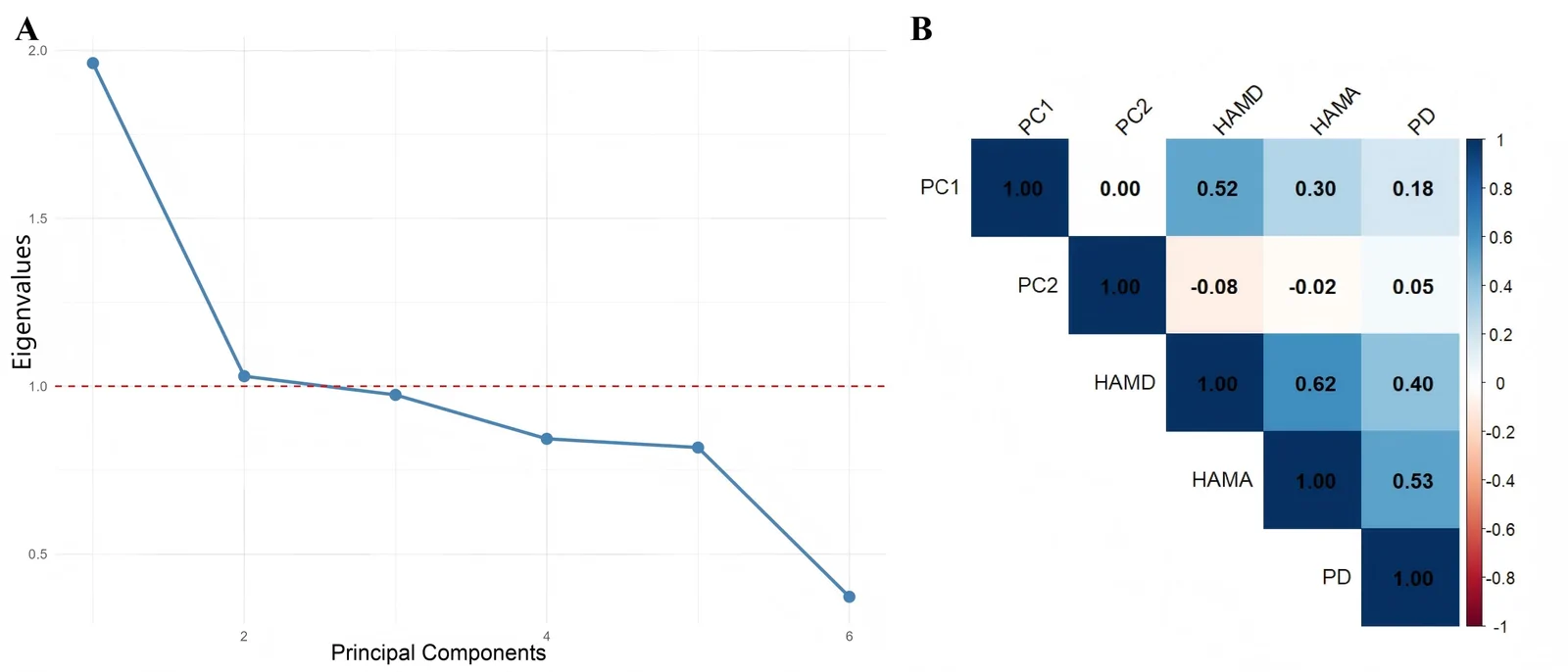
Extraction of metabolic principal components and their correlations with clinical symptoms. **(A)** Scree Plot of principal component analysis for metabolic indicators with eigenvalue-based component selection. The x-axis represents the extracted principal components (PC1 to PC6), and the y-axis represents the eigenvalue of each principal component. The blue line with dots illustrates the decreasing trend of eigenvalues across principal components. The red dashed horizontal line marks the threshold of eigenvalue = 1. Principal components with eigenvalues exceeding 1 (PC1 and PC2) were retained as valid components for subsequent mediation and correlation analyses. **(B)** Correlation heatmap of metabolic principal components and clinical variables. Pearson correlation heatmap of key variables among patients, including metabolic principal components (PC1, PC2), Hamilton Depression Scale (HAMD) scores, Hamilton Anxiety Scale (HAMA) scores, and psychotic depression (PD) status. The x-axis and y-axis both represent the included variables. Each colored cell displays the Pearson correlation coefficient **(r)** between two corresponding variables. The vertical color bar on the right serves as the scale legend: the gradient from blue to red indicates the magnitude of r, with blue representing positive correlations and red representing negative correlations. The correlation directions are consistent with the numerical values presented in **Supplementary Table 3**. HAMA, Hamilton anxiety rating scale; HAMD, Hamilton depression rating scale; PC1, principal component 1; PC2, principal component 2; PD, psychotic depression.

We identified two components, collectively explaining 49.87% of the total variance (**Supplementary Table 1**, **Figure 1A**).

The first principal component (PC1), designated the cholesterol-glucose metabolism component (eigenvalue = 1.9622, 32.7% variance explained), showed significant positive loadings on TC (0.5939), LDLC (0.5325), and FBG (0.3864), and a significant negative loading on HDLC (-0.3629) (**Supplementary Table 1, 2**, **Figures 1A, B**). Higher PC1 scores indicated dysregulated lipid metabolism and increased FBG levels.

The second principal component (PC2), designated the BMI-triglyceride metabolism component (eigenvalue = 1.0299, 17.17% variance explained), had significant positive loadings on TG (0.6984) and BMI (0.5110), and a significant negative loading on LDLC (-0.4333) (**Supplementary Table 1, 2**, **Figures 1A, B**). Higher PC2 scores reflected increased body mass and abnormal TG metabolism. LDLC showed opposing loading directions across the two principal components: positive in PC1 (0.5325) and negative in PC2 (-0.4333).

Bivariate Pearson correlation analysis revealed that all pairwise associations reached statistical significance (all P < 0.001) (**Supplementary Table 3**). PC1 was strongly positively correlated with HAMD (r = 0.5204), moderately correlated with HAMA (r = 0.3000), and weakly correlated with PD (r = 0.1812). Among clinical scales, HAMD and HAMA presented an extremely strong positive correlation (r = 0.6170). HAMD showed a moderate positive correlation with PD (r = 0.4034), whereas HAMA was strongly positively correlated with PD (r = 0.5340). PC2 showed no significant correlation with psychotic symptoms (r = 0.0256, P = 0.291). All these significant positive associations provided a solid basis for the subsequent chain mediation analysis.

The composite metabolic indicators derived from PCA were adopted for the chain mediation analysis and subsequent K-means clustering analysis.

### 3.3 Correlations of PANSS positive subscale with HAMD and HAMA

Pearson correlation analysis revealed that the PANSS positive subscale total score was significantly correlated with HAMD total score (r = 0.54, P < 0.001) and HAMA total score (r = 0.61, P < 0.001). The correlation between HAMD and HAMA total scores was 0.62 (P < 0.001).

At the item level, correlations with HAMD and HAMA total scores varied across the seven PANSS positive items. The core psychotic items, delusions and hallucinatory behavior, showed moderate associations with both affective scales, with coefficients ranging from 0.41 to 0.55. Conceptual disorganization yielded correlations of 0.52 with HAMD and 0.55 with HAMA. Suspiciousness/persecution and hostility, which inherently carry partial affective loading, showed relatively higher correlations with HAMD (0.59 and 0.58, respectively) and HAMA (0.61 and 0.57, respectively). In contrast, excitement (P4) exhibited weak but statistically significant correlations with HAMD (r = 0.18, P < 0.001) and HAMA (r = 0.26, P < 0.001). Grandiosity (P5) showed minimal and non-significant correlations with both HAMD (r = 0.07, P = 0.280) and HAMA (r = 0.09, P = 0.146). All other correlations were statistically significant at P < 0.001 (**Table 2**).

**Table 2:** Correlation matrix of PANSS positive subscale (total score and items), HAMD, and HAMA.

Variables	HAMD	HAMA	PANSS positive total	Delusions	Conceptual disorganization	Hallucinatory behavior	Excitement	Grandiosity	Suspiciousness/persecution	Hostility
HAMD	1									
HAMA	0.62***	1								
PANSS positive total	0.54***	0.61***	1							
Delusions	0.42***	0.54***	—	1						
Conceptual disorganization	0.52***	0.55***	—	0.87***	1					
Hallucinatory behavior	0.41***	0.53***	—	0.96***	0.90***	1				
Excitement	0.18***	0.26***	—	0.10***	0.14***	0.09***	1			
Grandiosity	NA	NA	—	NA	NA	NA	NA	1		
Suspiciousness/persecution	0.59***	0.61***	—	0.84***	0.84***	0.84***	0.15***	NA	1	
Hostility	0.58***	0.57***	—	0.62***	0.67***	0.60***	0.18***	NA	0.81***	1

Note: All Pearson correlation coefficients were statistically significant at P < 0.001. Correlations involving the grandiosity item were not estimable due to limited item variance in the sample. Correlations between the PANSS positive total score and individual positive items were not conducted, as the total score is an aggregate of these items and the correlation would be mathematically redundant. HAMA, Hamilton Anxiety Rating Scale; HAMD, Hamilton Depression Rating Scale; NA, not applicable; PANSS positive, Positive and Negative Syndrome Scale positive subscale; ***, P < 0.001.

### 3.4 Cross-sectional path decomposition of covariance patterns

**Figure 2:**
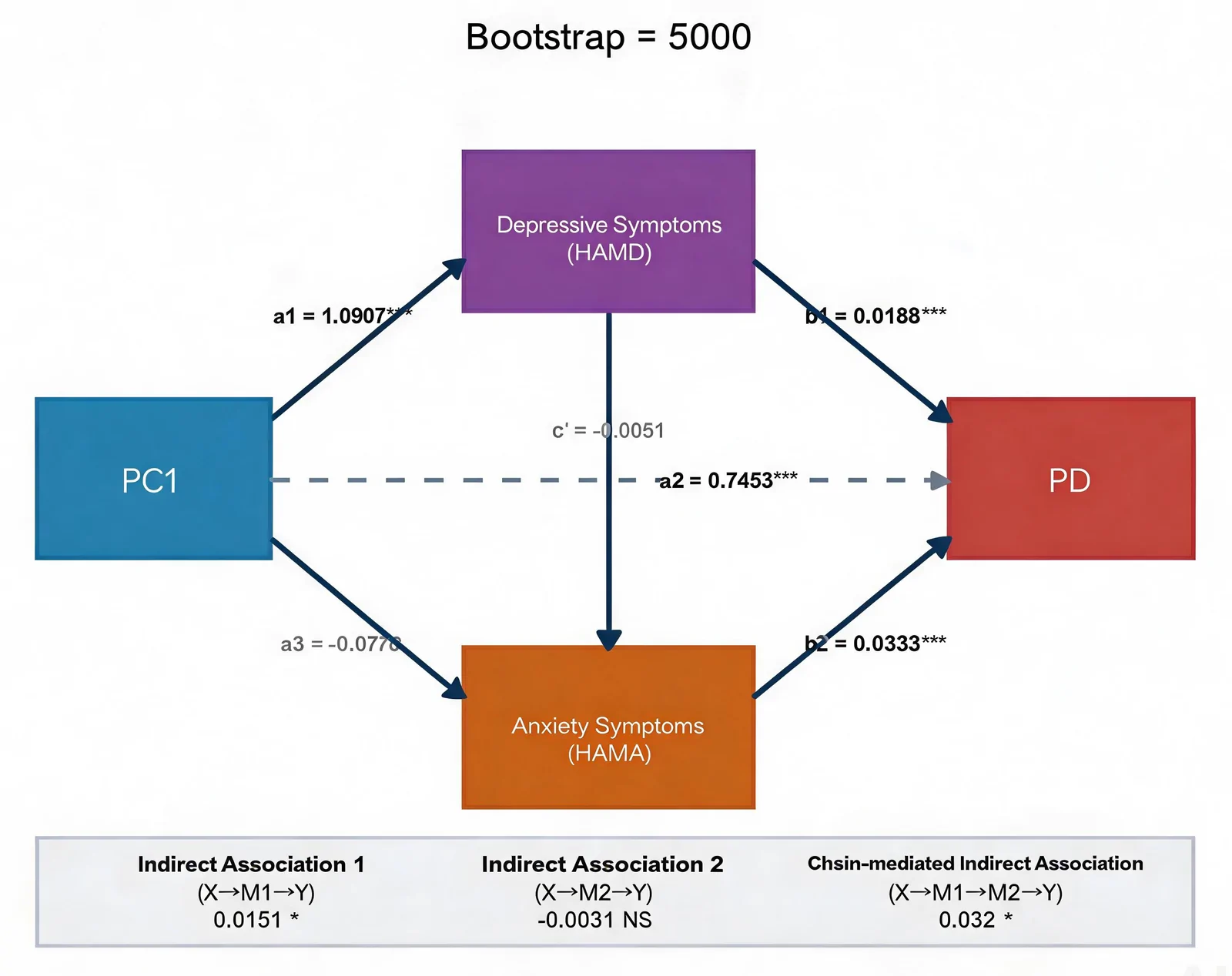
Serial association model of the association between metabolic dysregulation and psychotic symptoms. Bias-corrected bootstrap with 5000 resamples was used to test the significance of association effects. The independent variable (X) was metabolic principal component 1 (PC1, representing metabolic dysregulation), the sequential mediators were Hamilton Depression Scale (HAMD) score (M1) and Hamilton Anxiety Scale (HAMA) score (M2), and the dependent variable (Y) was psychotic depression (PD) status. Association coefficients are labeled on each path. All coefficients reflect associations derived from cross-sectional data. The table below presents the specific indirect associations: the indirect association via depressive symptoms (X→M1→Y) was significant, the indirect association via anxiety symptoms alone (X→M2→Y) was not significant, and the chain-mediated indirect association through both depressive and anxiety symptoms (X→M1→M2→Y) was significant. All coefficients in this figure reflect statistical associations derived from cross-sectional data. The terms **“**direct association**”**, **“**indirect association**”** and **“**sequential pattern**”** are used to describe the statistical structure of the data. The sequential ordering of variables represents the statistical model specification. a1: association of X with M1; a2: association of M1 with M2; a3: direct association of X with M2; b1: association of M1 with Y; b2: association of M2 with Y; c**’**: direct association of X with Y; HAMA, Hamilton anxiety rating scale; HAMD, Hamilton depression rating scale; NS, not significant; PC1, principal component 1; PD, psychotic depression; *, P < 0.05; ***, P < 0.001.

We found that PC1 was significantly positively associated with HAMD scores (a = 1.0907, 95% CI: 1.0122 - 1.1814), and HAMD scores were significantly positively associated with HAMA scores (a2 = 0.7463, 95% CI: 0.6969 - 0.7980) (**Supplementary Table 4**, **Figure 2**). Both HAMD (b1 = 0.0138, 95% CI: 0.0080 - 0.0210) and HAMA scores (b2 = 0.0393, 95% CI: 0.0342 - 0.0443) were significantly positively associated with the presence of psychotic symptoms, while PC1 showed no significant direct associations with HAMA scores (a3 = -0.0779, 95% CI: -0.1826 - 0.0325) or psychotic symptoms (c’ = -0.0061, 95% CI: -0.0192 - 0.0049).

The total association between PC1 and psychotic symptoms was 0.0379 (95% CI: 0.0267 - 0.0484) (**Supplementary Table 5**, **Figure 2**). The total indirect covariation component was significant (0.0440, 95% CI: 0.0361 - 0.0533), statistically explaining 116.1% of the total covariation between PC1 and psychotic symptoms. The single indirect association via depressive symptoms was significant (p = 0.0151, 95% CI: 0.0090 - 0.0231), accounting for 34.3% of the total indirect association. In contrast, the association pattern via anxiety symptoms alone was not significant (-0.0031, 95% CI: -0.0073 - 0.0014). Notably, the sequential chain-mediated association pattern via both mediators explained the largest proportion of the total association (0.0320, 95% CI: 0.0266 - 0.0384), contributing 72.7% of the total indirect association.

### 3.5 K-means clustering and metabolic risk stratification

**Figure 3:**
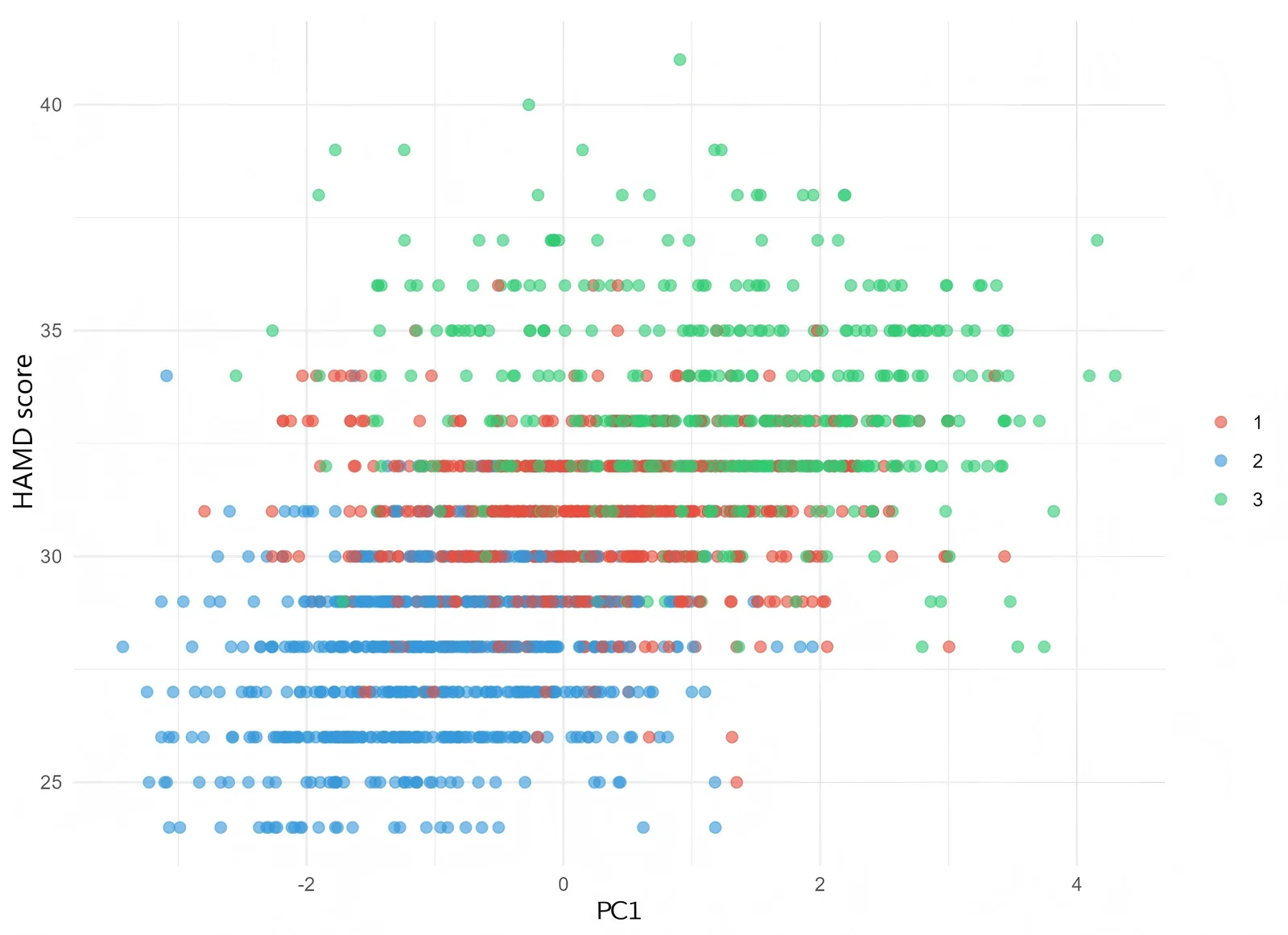
K-means clustering for metabolic risk stratification. The scatter plot stratifies patients using two core variables: metabolic indicator levels (represented by principal component 1, PC1) and depressive symptom severity (measured by the Hamilton Depression Scale, HAMD score). The x-axis represents PC1, reflecting the relative levels of metabolic indicators, and the y-axis represents HAMD scores, reflecting depressive symptom severity; each dot represents an individual patient, colored by their assigned cluster. Three risk-stratified groups were identified: low-risk (Cluster 2, blue dots, lower metabolic indicator levels and mild depressive symptoms), moderate-risk (Cluster 1, red dots, intermediate metabolic indicator levels and depressive symptoms), and high-risk (Cluster 3, green dots, higher metabolic indicator levels and severe depressive symptoms), providing distinct patient profiles for subsequent risk-related analyses. HAMD, Hamilton depression rating scale; PC1, principal component 1.

We identified three metabolic subgroups using K-means clustering, categorized as high, medium, and low risk groups based on PC1 scores and psychotic symptom risk (**Supplementary Table 6**, **Figure 3**). The high risk group (n = 453) had the highest PC1 score (1.1814), most severe depressive (HAMD = 33.36) and anxiety (HAMA = 24.59) symptoms, and a psychotic symptom prevalence of 32.23%. The medium risk group (n = 610) had an intermediate PC1 score (0.2121), moderate depressive (30.87) and anxiety (20.40) symptoms, with a prevalence of 3.44%. The low risk group (n = 655) had the lowest PC1 score (-1.0146), mildest depressive (27.65) and anxiety (18.54) symptoms, and the lowest prevalence at 0.61%.

All measured variables differed significantly across the three groups (all P < 0.001) with large effect sizes. We observed that the psychotic symptom prevalence in the high-risk group was approximately 52.8 times that in the low-risk group (crude prevalence ratio, unadjusted for confounders). This crude estimate should be interpreted with caution, as it does not account for potential confounding factors. Nonetheless, the clear gradient across the three groups (32.23% vs. 3.44% vs. 0.61%) supports a dose-response association between PC1-derived metabolic abnormalities and psychotic symptom prevalence.

### 3.6 ROC curve analysis

**Figure 4:**
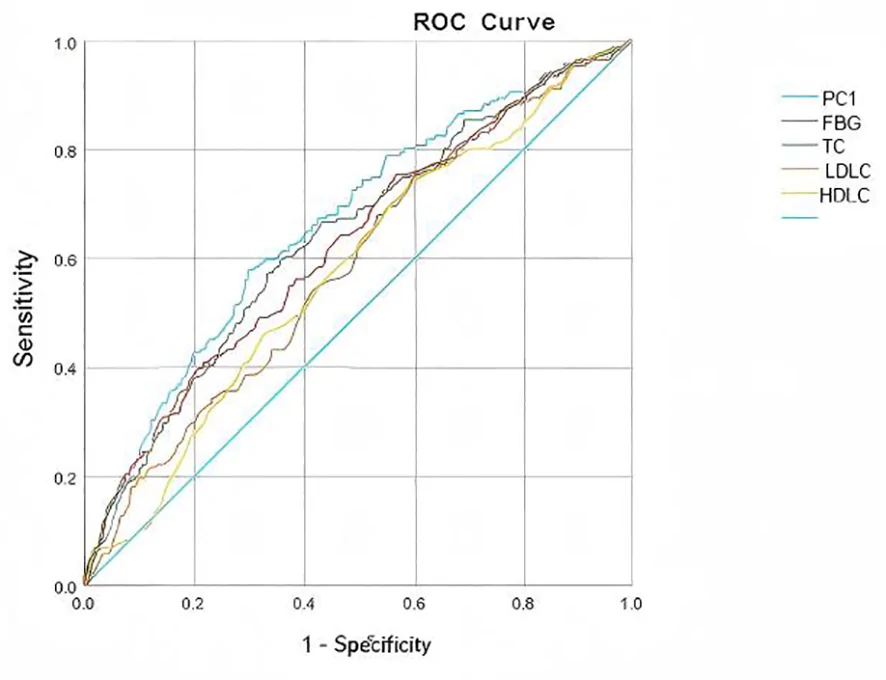
Receiver operating characteristic curve analysis for discrimination of psychotic symptoms. Receiver operating characteristic (ROC) curve analysis was conducted to evaluate the discriminative performance of metabolic indicators for psychotic symptoms. The y-axis denotes sensitivity, representing the true positive rate of correctly identifying cases with psychotic symptoms. The x-axis denotes 1 − specificity, corresponding to the false positive rate. Each colored line corresponds to a distinct marker: the cholesterol-glucose metabolic component (represented by principal component 1, PC1), fasting blood glucose (FBG), total cholesterol (TC), low-density lipoprotein cholesterol (LDLC), and high-density lipoprotein cholesterol (HDLC). The diagonal reference line reflects an area under the curve (AUC) of 0.5, indicating discriminative ability equivalent to random chance. PC1 yielded the highest AUC across all included markers, suggesting relatively stronger discriminative capacity compared with any single metabolic parameter. FBG, fasting blood glucose; HDLC, high-density lipoprotein cholesterol; LDLC, low-density lipoprotein cholesterol; PC1, principal component 1; ROC, receiver operating characteristic; TC, total cholesterol.

ROC analysis revealed that PC1 had an AUC of 0.664 (95% CI: 0.621 - 0.707), which was significantly higher than chance (P < 0.001). For individual metabolic parameters, the AUCs were 0.627 for FBG, 0.640 for TC, 0.588 for LDL-C, and 0.578 for HDL-C (all P < 0.01). PC1 demonstrated superior discriminative power compared to any single metabolic indicator (**Figure 4**).

### 3.7 Multivariate logistic regression analysis

We examined the independent association between metabolic risk subgroups derived from PC1-based K-means clustering and psychotic symptoms. Covariates included age, gender, education level, and marital status, selected based on univariate comparisons and clinical relevance, in accordance with the pre-specified statistical plan (**Table 3**). We found a strong independent association between metabolic risk stratification and psychotic symptoms. Compared with the high-risk metabolic subgroup, patients in the medium-risk subgroup had a 92.4% lower risk of psychotic symptoms (adjusted OR = 0.076, 95% CI: 0.047 - 0.122, P < 0.001). The protective effect was more pronounced in the low-risk subgroup, with a 98.7% reduced risk (adjusted OR = 0.013, 95% CI: 0.005 - 0.036, P < 0.001). The PC1 × sex interaction was not significant (P = 0.270). Stratified analyses showed comparable associations in males (OR = 1.305, 95% CI: 1.123 - 1.517, P = 0.001) and females (OR = 1.293, 95% CI: 1.185 - 1.411, P < 0.001), indicating no significant sex difference in this association. No significant independent associations were observed between age, gender, education level, marital status, and psychotic symptoms in the fully adjusted model (all P > 0.05). Collinearity diagnostics confirmed that all VIF values in the final model were below 5, indicating no substantial multicollinearity among the included covariates.

**Table 3:** Multivariate logistic regression for metabolic risk and psychotic symptoms.

Variable	β	SE	P	OR	OR_CI_lower	OR_CI_upper
X2	-2.582	0.245	<0.001	0.076	0.047	0.122
X3	-4.341	0.512	<0.001	0.013	0.005	0.036
age	0.01	0.011	0.345	1.01	0.989	1.032
sex (female)	0.324	0.201	0.106	1.383	0.933	2.05
edu (high school)	-0.375	0.238	0.115	0.687	0.431	1.095
edu (college)	-0.482	0.289	0.096	0.617	0.35	1.089
edu (postgraduate)	-0.697	0.449	0.121	0.498	0.206	1.202
marriage (married)	-0.462	0.279	0.097	0.63	0.365	1.087

X2 and X3 representing the medium-risk and low-risk metabolic subgroups. The high-risk metabolic subgroup derived from K-means clustering of retained principal components was set as the reference category. edu, education level; OR, adjusted odds ratio; OR_CI_lower, odds ratio confidence interval lower bound; OR_CI_upper, odds ratio confidence interval upper bound; SE, standard error; β, β coefficient.

## 4 Discussion

Using a large cohort of treatment-naïve patients with MDD, we observed that patients with PD exhibited distinct cholesterol and glucose metabolic profiles compared with those without psychotic symptoms. Serial association analysis revealed that the association between the cholesterol-glucose metabolic component (PC1) and psychotic symptoms was primarily indirect, mediated sequentially through depressive and anxiety symptoms. Risk stratification based on PC1 identified a clear dose-response gradient, with psychotic symptom prevalence increasing from 0.61% in the low-risk group to 32.23% in the high-risk group. These associations remained robust after adjusting for demographic confounders. Focusing on first-episode, treatment-naïve patients minimized confounding specifically related to prior antipsychotic or antidepressant exposure, a major concern in psychiatric metabolic research, but does not eliminate other potential confounders (e.g., somatic medications, diet, physical activity, and subclinical episodes). These empirical findings provide a foundation for understanding the metabolic correlates of this severe depressive subtype, and serve as a basis for the hypothesis-generating mechanistic considerations discussed below.

Depressive and anxious symptoms overlap substantially in patients with MDD. We found that correlations between the PANSS positive subscale and affective symptom scales did not exceed this reference level. This may support the interpretation that the positive subscale is not merely a proxy for overall illness severity. The subscale instead appears to retain variance specific to psychotic features, beyond what it shares with affective symptoms. Item-level findings align with this interpretation. Core psychotic items showed only moderate associations with affective symptoms. Items with greater affective overlap approached the HAMD-HAMA baseline correlation. This suggests shared variance stems partly from individual items, rather than the full subscale acting as a uniform marker of severity. Excitement showed weak associations with affective symptoms, consistent with its nature as a nonspecific activation symptom. Grandiosity showed weak associations with affective symptoms, consistent with its largely psychotic nature. This variability across items suggests the PANSS positive subscale captures a multidimensional construct rather than a unidimensional severity index. Psychotic and affective components overlap but remain partially distinct.

We examined five glycolipid markers together with BMI. Multiple metabolic markers differed notably in patients with PD. Individual measures showed strong collinearity, however, and could not capture the underlying metabolic pattern. To address this, we used PCA to reduce dimensionality and extract core metabolic dimensions. A distinct association emerged between the cholesterol-glucose dimension and PD, while the BMI-triglyceride dimension showed no significant link. This lack of association likely reflects counteracting effects within the latter dimension: triglycerides and BMI had positive loadings, low-density lipoprotein cholesterol had a negative loading, and these opposing influences offset one another.

In this study, we used PCA to identify a typical metabolic dimension dominated by cholesterol and glucose. It is plausible that cholesterol-glucose dysregulation may be linked to psychotic depression through systemic inflammation. Lipid and glucose metabolism interact closely in the body, and their imbalance is associated with systemic inflammation (21). Metabolic disorders are linked to inflammation that can cross the blood-brain barrier and activate cerebral microglia (22), which may then be associated with widespread neuroinflammatory responses. Such reactions have been proposed to disrupt the synthesis and balance of serotonin and dopamine in brain tissues (22). Altered tryptophan metabolism may lower serotonin bioavailability and cause neurotoxic metabolite buildup in the brain (22). Lipid metabolic abnormalities have also been linked to worsened neural pathological damage (23). Chronic stress related to psychotic symptoms may intensify central inflammation and form a theoretical pathological chain of inflammatory response, neurotransmitter disorder, and mental performance abnormality (24). Metabolic inflammatory cascades could plausibly link cholesterol-glucose disorders with psychotic symptoms, partly explaining the significant correlation observed in our research.

One speculative hypothesis is that cholesterol and glucose dysregulation might affect brain structure and synaptic function. Abnormal cholesterol metabolism has been associated with impaired brain structure and neuronal function, which may potentially relate to psychotic symptoms (25). Disturbed glucose metabolism interacts with lipid disorders, and their combined effect tends to be associated with aggravated central nervous system damage. Changes in cholesterol levels may affect hippocampal morphology, and elevated serum cholesterol is likely correlated with reduced hippocampal volume (26). Patients with psychotic depression tend to have smaller gray matter volume of the amygdala and hippocampal complex compared with individuals diagnosed with non-psychotic depression (27). Peripheral metabolic imbalance may disrupt neuronal cholesterol homeostasis, which can lead to abnormal dendrite growth, synaptic loss, and irregular neuronal activity in preclinical models (28). Lipid metabolic disturbance has been proposed to disrupt synaptic structure and interfere with signal transmission related to synaptic plasticity (23). Sustained hyperglycemia can trigger oxidative stress and the accumulation of toxic metabolites, which may impair the emotional regulatory brain regions (29). Metabolic imbalance may also disrupt endocrine stability, forming a deteriorating physiological cycle (30, 31). Based on this theoretical analysis, we presume that cholesterol and glucose disorders together may be associated with impaired central nervous function at multiple levels. This could explain why only the cholesterol-glucose dimension shows an evident correlation with psychotic depression.

Cholesterol and glucose play essential physiological roles in maintaining regular neural activities of the central nervous system. Cholesterol participates in neuronal membrane formation and myelin wrapping, and astrocytes modulate its synthesis, transport, and metabolism in brain tissues (32). Variations in cholesterol metabolism may directly affect normal neural physiological activities (33). Glucose provides fundamental energy for neuronal operations, and an insufficient energy supply may hinder regular brain function (34). Stable lipid and glucose metabolism may sustain synaptic transmission efficiency and individual cognitive capacity (35). Metabolic disturbance may lead to widespread neural dysfunction, supporting the notion that the cholesterol-glucose metabolic characteristic could serve as a potential biological marker for psychotic depression.

Another hypothesis-generating possibility relates to shared genetic underpinnings. The onset of psychotic depression may relate to shared genetic modulation of metabolic pathways and synaptic plasticity. Multiple overlapping genetic variants exist between schizophrenia and depression, and these risk genes tend to participate in synaptic function regulation (36). Genetic risk networks of major depressive disorder may have close connections with lipid metabolism, and core genes can regulate lipid metabolism via astrocyte-mediated processes (37). Metabolic regulatory genes may share overlapping segments with synaptic plasticity-related genes. Major depressive disorder may have a genetic correlation with cholesterol-glucose metabolic abnormality, and the two conditions may share partial core genetic pathways (38). Relevant genome studies indicate that lipid metabolic genes may cooperate with neurodevelopment genes to influence disease progression (39). Risk genes of psychotic depression may partially overlap with metabolic and synaptic regulatory pathways, potentially modulating brain cholesterol synthesis and glucose transport. Cholesterol-glucose metabolic characteristics may be inherent biological traits of psychotic depression. Our study results may provide partial evidence for this speculation. However, these genetic and mechanistic interpretations were not directly tested in our study and require independent validation in future mechanistic or longitudinal investigations.

LDLC exhibited opposing loadings across the two metabolic dimensions, which explains why only PC1 was associated with psychotic depression. In PC1, LDLC correlated positively with total cholesterol and fasting glucose to form a PD-linked metabolic profile, whereas in PC2, LDLC correlated inversely with BMI and triglycerides. This divergent pattern reflects the context-dependent metabolic role of LDLC. The positive alignment of LDLC with TC and FBG in PC1 represents a pro-atherogenic lipid-glucose axis. In PC2, the negative loading of LDLC alongside positive BMI and TG loadings matches the metabolic profile of obesity-related insulin resistance, where increased hepatic VLDL production elevates TG while LDLC levels may not rise proportionally or even decline due to altered lipoprotein metabolism (40, 41). This divergence accounts for the selective association of PC1 with psychotic symptoms. PC1 captures a coherent pro-atherogenic metabolic pattern, while PC2 reflects a mixed metabolic state where opposing effects counterbalance each other and weaken its specific link to the outcome.

We designated PC1 as the cholesterol-glucose metabolic component. Its loading pattern, marked by elevated FBG, TC, and LDLC alongside reduced HDLC, represents a cluster of abnormalities widely recognized as hallmarks of atherogenic dyslipidemia and insulin resistance. This metabolic state has previously been linked to neuropsychiatric outcomes through neuroinflammatory and neuroendocrine pathways (22, 23), and this coherent signature supports PC1 as a proxy for a functionally relevant metabolic dimension (23, 29). The absence of association between PC2 and psychotic symptoms does not imply BMI or TG are irrelevant to depression. Instead, it suggests the metabolic signature specific to psychotic features is selectively captured by the cholesterol-glucose axis, while the BMI and TG dimension reflects a more general adiposity-related pattern that is not specific to this severe depressive subtype. The dual role of LDLC indicates PD-related metabolic disturbances arise from network-level dysregulation rather than isolated marker changes, confirming the cholesterol-glucose dimension as a core metabolic feature of the disorder and clarifying its metabolic basis.

The loading pattern of PC1, marked by elevated FBG, TC and LDLC alongside reduced HDLC, appears consistent with the metabolic phenotype previously associated with neuropsychiatric disorders via neuroinflammatory and neuroendocrine pathways (22, 23). Although PC2 explained a substantial proportion of metabolic variance, it presented a relatively heterogeneous loading profile, with positive loadings for TG and BMI and a negative loading for LDLC. This opposing pattern may reflect a mixed metabolic state such as obesity-related insulin resistance that likely carries lower specificity for the psychotic subtype of depression (40, 41). Consistent with this notion, PC2 showed no significant bivariate correlation with psychotic symptoms, lending further supportive evidence for the relative specificity of the cholesterol-glucose axis to psychotic depression.

The serial association analysis revealed that the association between metabolic abnormalities and psychotic symptoms was primarily explained by indirect pathways. No significant direct association was observed after accounting for depressive and anxiety symptoms. It is important to note that these results reflect the correlational structure of the data rather than causal mediation effects, as the cross-sectional design cannot confirm temporal sequences between variables. The sequential association between depressive and anxiety symptoms accounted for the largest proportion of the observed link, with the pathway involving depression alone reaching statistical significance, while the pathway involving anxiety alone did not.

We found the total indirect association slightly exceeded the total association, yielding a non-significant negative direct association. As a cross-sectional investigation, our serial association analysis captures co-occurring association patterns rather than temporally validated causal or sequential relationships. This pattern aligns with a competitive association framework, in which direct and indirect association patterns may act in opposite directions (42).

Statistically, this pattern predominantly reflects model-specific properties rather than a genuine biological effect. The low prevalence of psychotic symptoms in our cohort reduces estimation precision for the binary outcome, and sequential inclusion of correlated intermediate factors HAMD and HAMA may introduce residual collinearity that impairs decomposition of total association into direct and indirect components (43). The small excess of total indirect relative to total association is also a recognized phenomenon in cross-sectional mediation-type models with binary endpoints, typically driven by coefficient scaling and parameterization rather than substantive biological processes (42, 43). We thus interpret the non-significant negative direct association as a statistical observation rather than evidence of a true protective effect, and this null result does not confirm the absence of an effect, given the potential for a Type II error due to limited statistical power.

Prior work has linked favorable lipid and lipoprotein homeostasis to milder psychotic symptoms in other populations (44, 45), but our cross-sectional design cannot verify such causal pathways. A speculative hypothesis that certain metabolic states may correlate with less severe psychotic manifestations requires dedicated prospective testing. Importantly, this hypothesis is not supported by our non-significant direct association estimates, and the observed statistical pattern is more conservatively explained by the model-specific factors outlined above. Any biological interpretation remains highly tentative and requires independent validation in future mechanistic or longitudinal studies.

Previous studies have explored the associations among depression, anxiety, and psychotic symptoms, as well as the connecting role and neurobiological basis of anxiety. Depressive and anxious symptoms are strongly associated with psychotic symptoms, and anxious distress in major depressive episodes is closely related to psychotic features (46). Prospective epidemiological studies indicate that anxiety and depressive symptoms may be premorbid risk factors for psychotic disorders and are linked to the later occurrence of psychotic symptoms (47). Anxiety and depressive symptoms may be associated with psychotic experiences through the affective pathway (48). Network analysis also shows that anxiety symptoms may bridge psychotic experiences and depressive symptoms (49). Patients with MDD combined with anxiety are more likely to present psychotic symptoms. FEDN MDD patients with anxiety have lower regional homogeneity in the right parahippocampal gyrus and more extensive abnormal spontaneous brain activity, which may form a vulnerable basis for psychotic symptoms (50). Anxiety-related prefrontal dysregulation may worsen patients’ perceptual and reality testing biases (51). Patients with anxious depression exhibit disrupted brain functional network connectivity related to altered peripheral transcriptional expression, and the related pathways are mainly involved in immune regulation (52). Elevated anxiety may be associated with brain network disorders through immune-related mechanisms and further linked to the occurrence of psychotic symptoms (52).

Most previous studies focused on the associations between emotional symptoms and psychotic symptoms and their biological mechanisms, but the roles of potential upstream factors and emotional links in theoretical pathogenic pathways lack a systematic explanation. Using mediation analysis, we further show that the association between metabolic abnormalities and psychotic symptoms is primarily explained by the sequential co-occurrence of depressive and anxiety symptoms. Our findings provide a new perspective for understanding the role of anxiety in PD. In this model, anxiety did not show an independent association with psychotic symptoms after adjusting for depression, indicating that it may act as a secondary factor accompanying more severe depressive symptoms in metabolically dysregulated patients. This does not rule out the possibility that anxiety may play an independent role in other subtypes of PD or in different patient populations. Multivariate regression confirmed that metabolic risk remained a significant independent predictor of psychotic symptoms after adjusting for variables, suggesting that potential demographic moderating effects are not strong enough to negate the overall association. Although women were overrepresented in the psychotic symptom group, the interaction between sex and the metabolic component was not significant, and stratified ORs were comparable between sexes, suggesting that sex did not materially modify the observed metabolic-psychosis association. Future larger multi-center studies should explore these moderating effects to refine the risk stratification model.

Current identification of psychotic symptoms in depression relies primarily on subjective rating scales with limited early detection sensitivity. Psychotic symptoms are often occult and intermittent, making them frequently overlooked. Delusions, in particular, may be misclassified as common depressive cognitions by less experienced clinicians (53). Although newer transdiagnostic scales cover broader symptom domains, they still rely on subjective assessment and lack objective biological correlates (54). Importantly, while metabolomics has shown promise for depression subtyping and treatment outcome prediction (55). Our findings suggest that metabolic risk stratification using these routine fasting glucose and lipid measurements offers an objective complementary approach to help distinguish subgroups of FEDN MDD patients with substantially different psychotic symptom risks.

We further validated the association between metabolic risk stratification and psychotic symptoms using multivariate logistic regression models. We adjusted all models for predefined demographic confounding factors. These factors included age, gender, education level and marital status. We found that metabolic risk level remained a significant independent predictor of psychotic symptoms in patients with MDD. Compared with the high metabolic risk subgroup, patients in the medium and low-risk subgroups showed a significantly lower risk of psychotic symptoms. These results confirm that the metabolic profile reflected by PC1-based metabolic risk stratification is an independent predictor of psychotic symptoms in depressed patients, rather than a confounding result caused by demographic factors.

In this study, we enrolled FEDN MDD patients to minimize confounding from medication exposure and prolonged disease course. With comprehensive statistical strategies and standardized quality control, we reduced research bias and improved the credibility and applicability of our findings. Our study provides new insights into the biological correlates and clinical management of psychotic depression. It deepens the understanding of metabolic features associated with this disorder, supports the use of the metabolic risk score for psychotic symptom risk stratification. Our findings provide a rationale for future prospective studies investigating whether maintaining glucose and lipid metabolic health is associated with a lower prevalence of psychotic depression.

The ROC analysis further substantiates the discriminative value of the cholesterol-glucose metabolic component. The AUC of 0.664 for PC1, though moderate, consistently exceeded that of any single glycolipid marker, indicating that the composite metabolic dimension captures risk-related information beyond individual parameters. This moderate accuracy suggests that while PC1-derived metabolic stratification is useful for identifying higher risk subgroups, it should be interpreted as a complementary screening tool rather than a definitive prognostic test. These findings align with the multifactorial nature of psychotic depression, where no single biological marker is expected to achieve perfect discrimination.

Our study has several limitations. First, the cross-sectional design only allows us to identify associations between variables and cannot establish causal or temporal relationships. Lacking onset timing data, reverse causality cannot be excluded, our path model is descriptive, as recall was unreliable in this acute cohort. Longitudinal cohort studies are needed to confirm the sequence of metabolic abnormalities, affective symptoms and psychotic symptoms. Additionally, duration of the current depressive episode was not compared between groups and may confound the observed associations. Second, the single-center Han Chinese sample limits the generalizability of our findings, and multi-center studies across different populations are required for validation. Third, we only examined basic glycolipid markers and BMI, and future studies should include inflammatory factors and other metabolic indicators to further explore the underlying mechanisms. Fourth, we did not formally test whether PC2 moderates the association between PC1 and psychotic symptoms, a potential interaction that warrants further investigation in larger samples. Fifth, the K-means clustering was exploratory and requires validation in independent cohorts. We also lacked data on somatic medications, diet, physical activity, and prior subclinical episodes. Our exploratory PANSS cutoff of 15 lacks clinical validation, and the benchmarking-derived interpretation requires replication or external validation in independent samples.

## 5 Conclusions

Our study demonstrates a significant association between cholesterol-glucose metabolic dysregulation and psychotic symptoms in FEDN MDD patients. Fasting glucose and lipid profiles may serve as complementary markers for identifying subgroups of patients with differing psychotic symptom prevalence. Our findings provide empirical evidence to inform future research and clinical assessment of psychotic depression. Prospective longitudinal studies with systematic documentation of symptom emergence chronology are urgently needed to resolve the directionality of these associations.

## Data Availability

The raw data supporting the conclusions of this article will be made available by the authors, without undue reservation.
